# Structure Determination of Binuclear Triple-Decker Phthalocyaninato Complexes by NMR via Paramagnetic Shifts Analysis Using Symmetry Peculiarities

**DOI:** 10.3390/molecules27227836

**Published:** 2022-11-14

**Authors:** Sergey P. Babailov, Eugeny N. Zapolotsky, Eduard S. Fomin, Marina A. Polovkova, Gayane A. Kirakosyan, Alexander G. Martynov, Yulia G. Gorbunova

**Affiliations:** 1 Nikolaev Institute of Inorganic Chemistry, The Siberian Branch of the Russian Academy of Sciences, Av. Lavrentyev 3, 630090 Novosibirsk, Russia; 2 Institute of Cytology and Genetics of the Siberian Branch of the Russian Academy of Sciences, Av. Lavrentyev 10, 630090 Novosibirsk, Russia; 3 Frumkin Institute of Physical Chemistry and Electrochemistry of the Russian Academy of Sciences, Leninskii pr. 31-4, 119071 Moscow, Russia; 4 Kurnakov Institute of General and Inorganic Chemistry, Russian Academy of Sciences, Leninskii pr. 31, 119991 Moscow, Russia

**Keywords:** NMR spectroscopy, lanthanides, phthalocyanines, symmetry, pseudo-contact contribution of lanthanide-induced shifts

## Abstract

The detailed knowledge about the structure of multinuclear paramagnetic lanthanide complexes for the targeted design of these compounds with special magnetic, sensory, optical and electronic properties is a very important task. At the same time, establishing the structure of such multinuclear paramagnetic lanthanide complexes in solution, using NMR is a difficult task, since several paramagnetic centers act simultaneously on the resulting chemical shift of a particular nucleus. In this paper, we have demonstrated the possibility of molecular structure determination in solution on the example of binuclear triple-decker lanthanide(III) complexes with tetra-15-crown-5-phthalocyanine Ln_2_[(15C5)_4_Pc]_3_ {where Ln = Tb (**1**) and Dy (**2**)} by quantitative analysis of the pseudo-contact lanthanide-induced shifts (LIS). The symmetry of complexes was used for the simplification of the calculation of pseudo-contact shifts on the base of the expression for the magnetic susceptibility tensor in the arbitrary oriented magnetic axis system. Good agreement between the calculated and experimental shifts in the ^1^H NMR spectra indicates the similarity of the structure for the complexes **1** and **2** in solution of CDCl_3_ and the structure in the crystalline phase, found from the data of the X-ray structural study of the similar complex Lu_2_[(15C5)_4_Pc]_3_. The described approach can be useful for LIS analysis of other polynuclear symmetric lanthanide complexes.

## 1. Introduction

Lanthanide complexes with phthalocyanine ligands exhibit unique physical and chemical behavior [1,2,3] responsible for their application as components of electrochromic materials and sensors [4,5], molecular switches and memory devices [6,7], fluorescent probes [8], single molecule magnets [9] and MRI contrasting agents [10]. Another promising application of those complexes is clinical magnetic resonance tomography, which can simultaneously perform magnetic imaging and measure 3D temperature distributions. The accurate non-invasive local temperature measurements in living cells is a crucial task for modern medicine and biochemistry [11,12,13,14]. For these approaches, lanthanide-containing compounds and complexes are very promising [15,16,17]. Targeted design of such materials requires the investigation of the relationships between the structure of lanthanide complexes and their functional properties [18]. Nuclear magnetic resonance spectroscopy (NMR) is a very efficient tool for studying the molecular structure, paramagnetic properties and molecular dynamics of paramagnetic lanthanide complexes. Thus, methods for the structure determination of mononuclear lanthanide complexes, based on the analysis of paramagnetic lanthanide-induced shifts (LIS), are successfully applied for both small molecules and biological systems containing proteins and nucleic acids [19,20,21,22,23,24,25,26,27,28,29,30,31].

**Figure 1:**
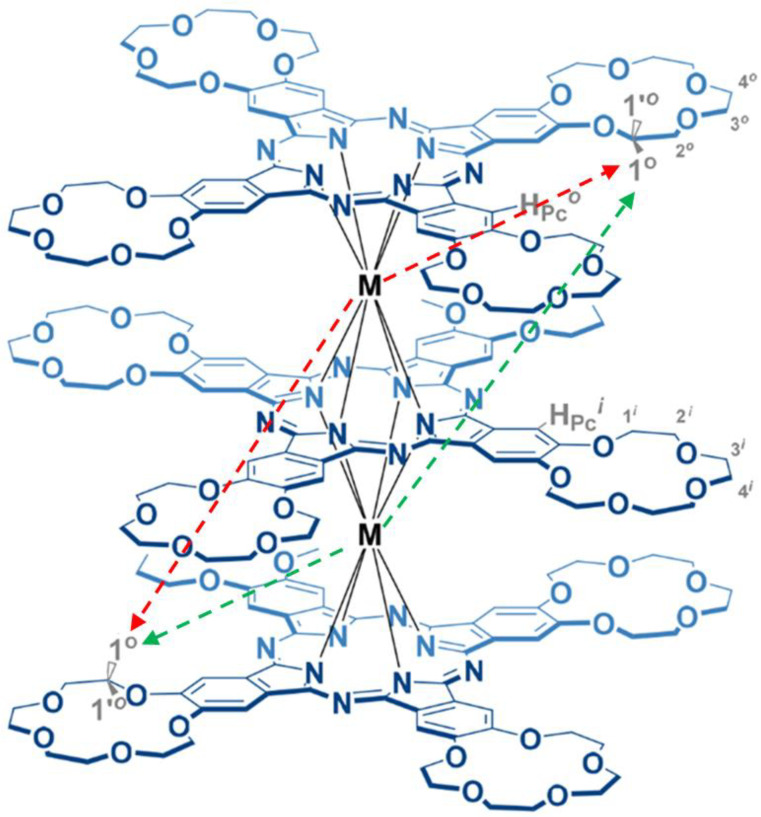
Schematic representation of the structure of the complexes **1** and **2** with an example of the arrangement of “paired” protons (where M = Tb (**1**) or Dy (**2**)).

In the presence of a structural model for the system under study (obtained from the X-ray structural analysis data or obtained by quantum-mechanical modeling and other methods), it is possible to use the method for analyzing pseudo-contact contributions to the LIS, based on the optimization procedure [32,33,34,35,36,37,38,39]. At the same time, NMR studies of polynuclear lanthanide complexes are more difficult, since each paramagnetic center contributes to the LIS as well as the increase in the relaxation rate. Accordingly, the qualitative and quantitative analysis of the LISs of polynuclear complexes is significantly complicated due to the increase in the number of unknown parameters (determined, for example, by quantum-chemical calculations or X-ray diffraction studies) [39]. However, in some cases, one can try to simplify structural calculations, for example, due to the symmetry of the complex. In this work, we use the method of simplifying the structural calculations of tetra-15-crown-5-phthalocyanine Ln_2_[(15C5)_4_Pc]_3_ {where Ln= Tb (**1**) and Dy (**2**)} taking into account their symmetry, Figure 1.

## 2. Results and Discussion

### Structural Assignment by NMR

**Figure 2:**
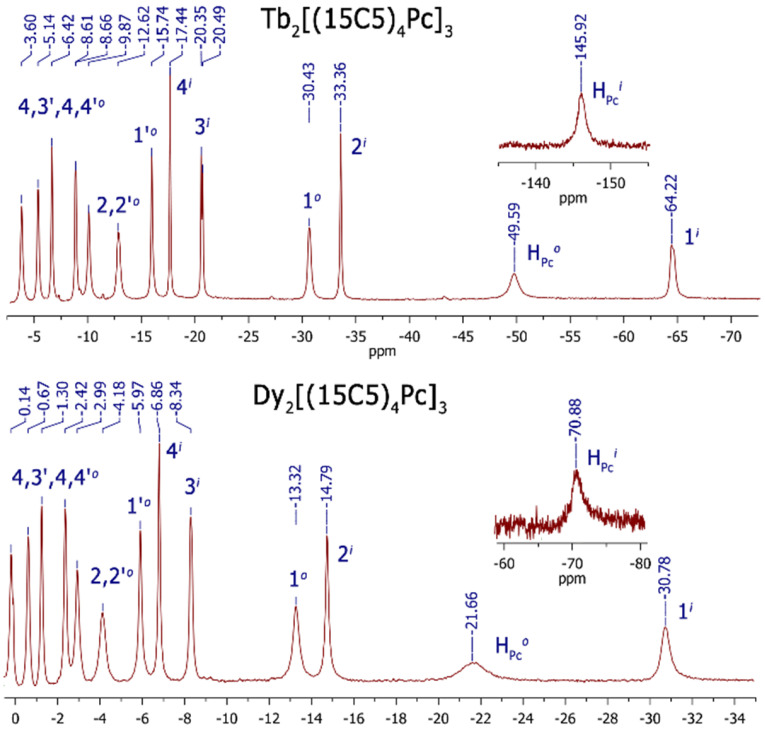
^1^H NMR spectra of Ln_2_[(15C5)_4_Pc]_3_ (where Ln = Tb, Dy) measured in CDCl_3_ at 303 K.

Previously, all observed signals in the ^1^H NMR spectra of Ln_2_[(15C5)_4_Pc]_3_ (Ln = Tb, Dy) (Figure 2) were assigned by complementary LIS and relaxation rate analysis with satisfactory convergence [40]. This analysis was made by simplified method (by «axial approximation»). The direct use of Formula (1) is limited by the complexity of the calculation procedures associated with an increase in the variable parameters. A more rigorous description of the pseudo-contact interaction for a polynuclear complex leads us to a more complex task. In our case, however, the calculations can be simplified using the symmetry of complexes.

In this work, we present a new approach for calculating paramagnetic pseudo-contact shifts in NMR spectra using the example of three-decker homobinuclear homoleptic complexes of lanthanides with phthalocyanine (**1** and **2**, Figure 1 and Figure 2). The complexes are characterized by the C_4h_ symmetry, and the plane of symmetry coincides with the inner phthalocyaninate deck. The outer decks (“upper” and “lower”) are mirror-like and inverted reflections of each other. Thus, each proton from the outer “upper” phthalocyaninate deck has its own “opponent” with a similar chemical and coordination environment in the “lower” deck (Figure 1). Each of the paramagnetic centers Ln^1^ and Ln^2^, interacting with a pair of “duplicating” (“paired”) protons from the “upper” and “lower” outer decks of phthalocyanine, and induces similar pseudo-contact shifts on them. Using such a symmetric arrangement of paramagnetic centers and “paired” protons, it is possible to simplify the computational task from the case of a binuclear complex to a quasi-mononuclear case (the detailed description of the calculation procedure is presented in Supplementary Materials).

The calculation of pseudo-contact LISs, carried out according to the procedure for a quasi-mononuclear complex (see detailed describing in Supplementary Materials), led to a set of theoretical values of *δ*_LIS_ (calc) for each of the complexes with an agreement factor of about 6% in both cases (see Table 1). The AF parameters were obtained as the optimization result {0.06 both for **1** and **2**}, confirming the consistency between the calculated and experimental LISs. The values of the calculated paramagnetic LIS for protons of different groups and the corresponding experimental LISs (Table 1) show a good convergence in these complexes. Indeed, the structure of the complexes **1** and **2** in the solution is consistent to the structure of single crystals of Lu_2_[(15C5)_4_Pc]_3_ obtained by single-crystal X-ray diffraction analysis.

The calculated values of the magnetic susceptibility tensor of the lanthanide cations are presented in Table 2. As can be seen from Table 2, the values of the parameters for the Tb complexes are approximately two times greater than those for the Dy complexes. This explains the observed trend that the paramagnetic LIS values for Tb complexes are about two times greater than for Dy complexes (see Table 2).

The observed little discrepancy between the calculated and experimental values of LISs (for H atoms) in complexes **1** and **2** can be due to several reasons. First, the Fermi-contact contribution to the LIS was not taken into account. Secondly, the cationic radius of Tb^3+^ and Dy^3+^ is about 5% larger than the cationic radius of Lu^3+^. Moreover, the influence of the solvate shell of the complexes, which can distort the spatial structure of complexes in solution compared to the crystal structure in solid state.

In addition, the “partial” contributions *Δδ*_LIS_ (Ln^1^) and *Δδ*_LIS_ (Ln^2^) of each of the lanthanides to the “total” shift *δ*_LIS_(calc) were calculated (see Table 1). These values may be useful for an in-depth understanding of the paramagnetic properties of the complexes.

Previously, the 2D distribution of the “zero” values of the paramagnetic pseudo-contact contributions of LIS for heteroleptic triple-decker symmetric two-nuclear lanthanide complexes of a similar structure have been calculated [38]. In that case, a simplified one-parameter expression was used to calculate the paramagnetic pseudo-contact contributions of the LIS. In addition, the authors assumed that the *z* axis of the paramagnetic susceptibility tensor coincides with the symmetry axis of the complexes (which may not always exist in specific systems) [38]. In particular, closed and open curves were revealed on 2D images of “zero” values of the paramagnetic pseudo-contact contributions of the LIS. Moreover, the orientation of the dumbbell-shaped distribution of the negative pseudo-contact contributions of the LIS coincides with the symmetry axis of the complex.

**Figure 3:**
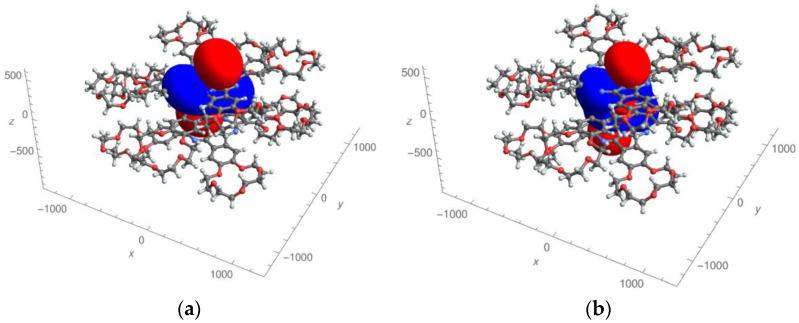
Effective field of the pseudo-contact contributions to lanthanide-induced shifts in the Dy complex created by only one lanthanide cation (**a**) and two lanthanide cations (**b**). Areas with positive shift values are shown in red, and areas with negative shift values are shown in blue.

**Figure 4:**
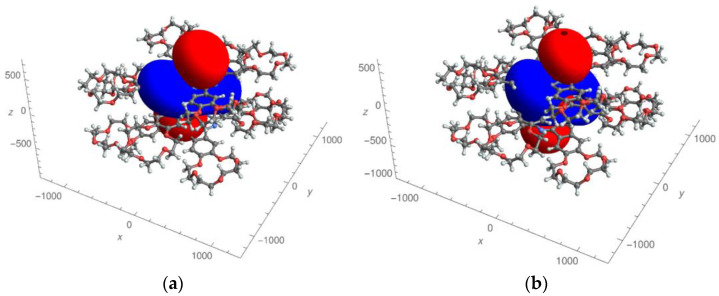
Effective field of pseudo-contact contributions to lanthanide-induced shifts in the Tb complex created by only one lanthanide cation (**a**) and two lanthanide cations (**b**). Areas with positive shift values are shown in red, and areas with negative shift values are shown in blue.

**Figure 5:**
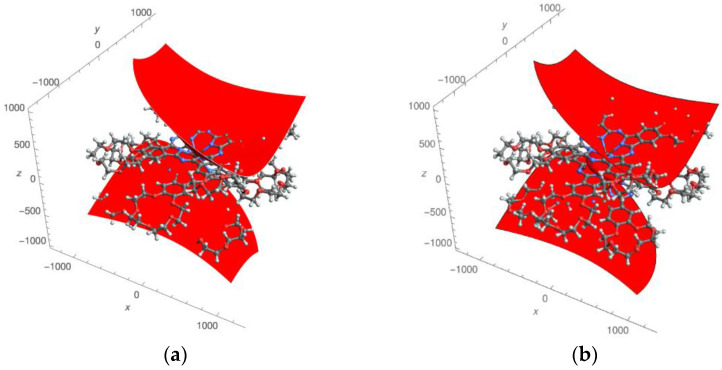
Two surfaces with zero values of pseudo-contact contributions to lanthanide-induced shifts created by only one paramagnetic cation (**a**) in the Dy complex; three surfaces with zero values of pseudo-contact contributions to lanthanide-induced shifts created by two paramagnetic centers (**b**).

In the presented work, we used the most complete (five-parameter) expression for the paramagnetic pseudo-contact contributions of the LIS, which is valid for an arbitrarily oriented coordinate system (see Formula (1)). There was no binding of the axes of the paramagnetic susceptibility tensor to the symmetry axis of the complexes. For the first time, 3D images of the distribution of pseudo-contact LISs (Figure 3 and Figure 4) and “zero” pseudo-contact LISs (Figure 5) in a paramagnetic binuclear lanthanide complex with identical ligands were constructed. In addition, partial 3D images of the distribution of “zero” pseudo-contact LISs from an individual paramagnetic center in a paramagnetic binuclear lanthanide complex were determined (Figure 5a). 

Further, in the text, we would like to consider and discuss the obtained results in more detail. The experimentally determined susceptibility tensor can be displayed in the form of pseudo-contact contributions to lanthanide-induced shift fields (see Figure 3 and Figure 4 for the Dy and Tb complexes, respectively). Figure 3a and Figure 4a show the distribution fields of chemical shifts created by one of the paramagnetic centers (in this case, the upper metal cations of the Dy and Tb complexes, respectively). Positive pseudo-contact shifts (PCSs) are shown in red, and negative PCSs are shown in blue. The method of presenting the calculation results is similar to that used in references [38]. The distribution of chemical shifts created by both paramagnetic centers are presented in Figure 3b and Figure 4b according to the same manner. 

It should be noted that the fields for the Tb complex visually differ from the fields for the Dy complex. A red «dumbbell» and a blue oval «collar» are visually observed (in the case of complexes with one paramagnetic center (Figure 3a and Figure 4a). In the case of two paramagnetic centers in both cases (Figure 3b and Figure 4b), a more complex figure is observed. Although the red “dumbbells” can be visually detected (in both cases, Figure 3b and Figure 4b), an additional “red” region is observed between the two paramagnetic centers.

The found values of the angles *θ* and *φ*, characterizing the orientation of the “dumbbells”, turned out to be equal: 18.5° and 36.3° for Dy and 18.9° and 37.7° (for Tb, correspondently). Here, the angle *θ* corresponds to the orientation of the “dumbbell” relative to the *z*-axis (coinciding with the axis passing through the Ln cations); *φ* is the angle of rotation of the dumbbell axis around the *z* axis (*φ* = 0 if the projection of the dumbbell axis on the *xy* plane coincides with the *x* axis). The presence of dumbbell-shaped distributions of positive and negative LIS values is generally consistent with [39]. The fact that the angle *θ* for complexes **1** and **2** is nonzero is an important distinguishing feature compared to the results of [41].

For the first time, we analyzed 3D surfaces with a zero value of PCSs. As can be seen, in the case of complexes with one paramagnetic center, there are two surfaces (Figure 5a). In the case of two paramagnetic centers, there are three surfaces (as seen in Figure 5b). Two of them are open, and one, located between the paramagnetic centers, has a closed character. This result, on the one hand is in agreement with previously found peculiarities, and, on the other hand, it is a generalization of the result obtained in reference [41] (where 2D images of the distribution of “zero” pseudo-contact shifts in the NMR spectra are given).

Quite similar calculation results were obtained in the analysis of chemical shifts in the terbium complex (Supplementary Materials).

In the work of Ishikawa [41], for the analysis of LIS on ligand protons in binuclear three-deck complexes, related to those presented in Figure 3, a simplified one-parameter analytical expression was used under the assumption that the axis of the paramagnetic susceptibility tensor associated with each of the two paramagnetic metal centers coincides with the axis of symmetry complexes. The authors of [41] described in 2D the distribution of positive and negative LISs in space (a kind of “dumbbells”), as well as lines describing zero LISs. We have solved this problem in the most general form (using five parametric expressions for LIS ) in an arbitrary coordinate system using the example of complexes shown in Figure 3. Using the symmetry of the complex, the problem was reduced from a “two-center” problem to a “one-center” problem without loss of generality. As a result of 3D modeling, it was found that the axis of the paramagnetic susceptibility tensor (the red “dumbbell” characterizing positive LIS ) does not coincide with the axis of symmetry of the complex, but is shifted by 19 degrees. For the first time, the surfaces of “zero” LISs were calculated (Figure 3). It turned out that there is one closed surface and two non-closed surfaces of “zero” LIS (see Figure 3).

It can be noted that a simple formal analysis carried out by us, in a visual form, led to rather interesting results. It seems that the further approbation of this method is quite possible for the purpose of structural analysis and calculation of paramagnetic chemical shifts of a wide range of compounds based on symmetric polynuclear lanthanide complexes.

## 3. Materials and Methods

### 3.1. Materials and Equipment

The complexes **1** and **2** as well as the diamagnetic counterpart Y_2_[(15C5)_4_Pc]_3_ were synthesized in moderate yields (50%, approx.) using a previously described procedure [40]. 

^1^H NMR spectra were recorded on a Bruker Avance III spectrometer operating at 600 MHz in CDCl_3_ in the presence of 10 µL of a 1% solution of N_2_H_4_·H_2_O in CD_3_OD and at ambient temperature with the use of the residual solvent resonance as internal reference.

### 3.2. Paramagnetic NMR Shifts Analysis

**Figure 6:**
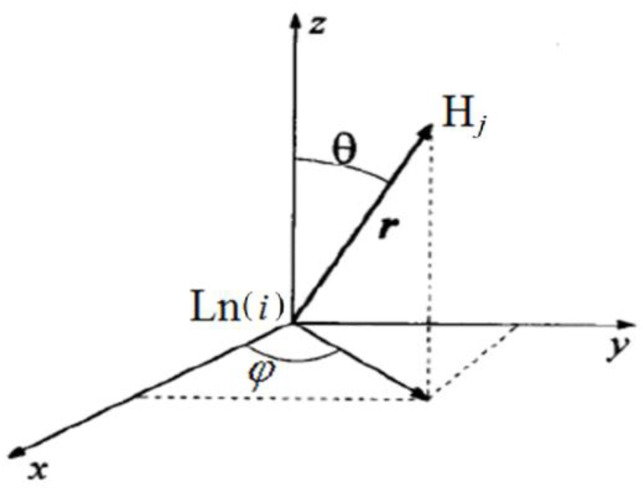
Spherical coordinates *r*, *θ*, *φ* of the nucleus of the hydrogen atom H_j_ relative to the cation Ln, given in Formula (1).

A commonly used approach of the LIS analysis is based on expression in the arbitrary magnetic axis system. The pseudo-contact contribution of LIS (in ppm) for a mononuclear complex can be expressed in the most general form through the tensor of molar magnetic susceptibility *χ* [25,42,43]:

(1) $$\delta_{j}^{PC}=\frac{1}{2N\hbar \gamma }\left[\begin{array}{l} \left(\bar{\chi }-\chi_{zz}\right)\langle \frac{1-3\cos^{2}\theta }{r^{3}}\rangle +\left(\chi_{xx}-\chi_{yy}\right)\langle \frac{\sin^{2}\theta \cos2\varphi }{r^{3}}\rangle +\\ +2\left(\chi_{xy}\right)\langle \frac{\sin^{2}\theta \sin\varphi }{r^{3}}\rangle +2\left(\chi_{xz}\right)\langle \frac{\sin2\theta \cos\varphi }{r^{3}}\rangle +2\left(\chi_{yz}\right)\langle \frac{\sin2\theta \sin\varphi }{r^{3}}\rangle \end{array}\right]$$

where *r*, *θ*, *φ* are the spherical coordinates of the nucleus relative to the Ln cation (the distance between the resonating nucleus of the hydrogen atom and the Ln cation is expressed in Å, Figure 6). Formula (1), which already contains five terms on the right side of the equation, is given for the case of an arbitrary chosen coordinate system, centered on the lanthanide ion.

In this work, a structural model based on the data obtained from X-ray structural analysis for the Lu_2_[(15C5)_4_Pc]_3_ complexes was used [44]. The coordinates of similar “paired” protons of phthalocyaninate decks, symmetrically located relative to the plane of symmetry, were removed from the structural model. Based on the structural data, the structural parameters were calculated for each set of paired protons corresponding to each other according to the procedure presented in Supporting Information (SI).

## 4. Conclusions

In summary, a new approach for calculating paramagnetic pseudo-contact shifts in NMR spectra was developed using the example of triple-decker homobinuclear homoleptic complexes of lanthanides with crown-phthalocyanine. Due to the symmetry of complexes, the calculation task for the binuclear complex has been reduced to the quasi-mononuclear case. The obtained values of the calculated paramagnetic LIS for the protons of different groups and the corresponding experimental LISs shows a good correlation. The distribution fields of chemical shifts created by both paramagnetic centers have a complex form compared to the one lanthanide case.

The results of the LISs analysis obtained in this work are a 3D generalization of the data obtained from the 2D analysis of the LISs by Ishikawa et al. for other substituted heteroleptic triple-decker phthalocyanine complexes. It turned out, in particular, that the orientation of the dumbbell-shaped distributions of positive LIS in the studied complexes did not coincide with the orientation of the symmetry axis (which was assumed in Ishikawa’s work), but were shifted by about 19° with respect to the symmetry axis of the complexes. The approach described here can be useful for LIS analysis of other polynuclear symmetric lanthanide complexes.

## Figures and Tables

**Table 1:** The observed *δ*_LIS_ (exp, ppm) and calculated *δ*_LIS_ (calc, ppm) pseudo-contact contributions to lanthanide-induced shifts in the ^1^H NMR spectra of the Ln_2_[(15C5)_4_Pc]_3_ (Ln = Tb and Dy) complexes in CDCl_3_ at 303 K.

Assignment	Tb				Dy			
	*δ*_LIS_(exp)	*δ*_LIS_(calc)	*Δδ*_LIS_ (Ln^1^)	*Δδ*_LIS_ (Ln^2^)	*δ*_LIS_(calc)	*δ*_LIS_(exp)	*Δδ*_LIS_ (Ln^1^)	*Δδ*_LIS_ (Ln^2^)
H_Pc_^o^	−57.6	−56.6	−71.4	14.8	−29.2	−29.7	−36.8	7.6
1^o^	−37.9	−35.4	−32.6	−2.79	−18.1	−19.4	−16.8	−1.4
1′^o^	−25.0	−25.4	−28.8	3.4	−13.1	−12.9	−14.9	1.8
2^o^	−21.9	−23.2	−18.7	−4.5	−12.0	−11.4	−9.6	−2.3
2′^o^	−20.3	−18.2	−15.7	−2.5	−9.4	−10.4	−8.1	−1.3
3^o^	−12.6	−14.0	−10.1	−3.8	−7.1	−6.4	−5.1	−1.9
3′^o^	−10.4	−16.3	−11.9	−4.3	−8.4	−5.3	−6.1	−2.2
4^o^	−9.0	−10.5	−8.9	−1.6	−5.3	−4.5	−4.5	−0.8
4′^o^	−7.6	−13.4	−11.3	−2.1	−6.9	−3.8	−5.8	−1.0
H_Pc_^i^	−154.3	−153.6	−150.2	−3.4	−79.0	−79.3	−78.9	−0.2
1^i^	−69.5	−67.7	−55.4	−12.3	−34.8	−35.9	−28.9	−5.9
2^i^	−35.1	−37.6	−28.1	−9.5	−19.4	−18.2	−14.7	−4.7
3^i^	−16.9	−15.1	−13.2	−1.9	−7.5	−8.5	−6.8	−0.8
4^i^	−14.0	−19.0	−14.8	−4.2	−9.7	−7.1	−7.7	−2.0
Sqrt(AF)	0.06				0.06			

**Table 2:** Calculated values of the components of the magnetic susceptibility tensor of the Ln cation $\frac{\left(\bar{\chi }-\chi_{zz}\right)}{N\times \hbar \times \gamma }$, $\frac{\left(\chi_{xx}-\chi_{yy}\right)}{N\times \hbar \times \gamma }$, $\frac{\chi_{xy}}{N\times \hbar \times \gamma }$, $\frac{\chi_{xz}}{N\times \hbar \times \gamma }$, $\frac{\chi_{yz}}{N\times \hbar \times \gamma }$ (expressed in ppm × Å^3^) for the Ln_2_[(15C5)_4_Pc]_3_ complexes in CDCl_3_ at 303 K.

Ln	$\frac{\left(\bar{\chi }-\chi_{zz}\right)}{N\times \hbar \times \gamma }$	$\frac{\left(\chi_{xx}-\chi_{yy}\right)}{N\times \hbar \times \gamma }$	$\frac{\chi_{xy}}{N\times \hbar \times \gamma }$	$\frac{\chi_{xz}}{N\times \hbar \times \gamma }$	$\frac{\chi_{yz}}{N\times \hbar \times \gamma }$
Tb	−89,395	23,001	−29,816	−70,807	−66,432
Dy	−45,992	11,872	−16,379	−38,628	−34,892

## Data Availability

Not applicable.

## Supplementary Materials

The supporting information can be downloaded at: https://www.mdpi.com/article/10.3390/molecules27227836/s1.

## Sample Availability

Samples of the compounds are available from the authors.
