# Supplementary material for: Structure Determination of Binuclear Triple-Decker Phthalocyaninato Complexes by NMR via Paramagnetic Shifts Analysis Using Symmetry Peculiarities
Source: Molecules. 2022 Nov 14;27(22):7836. doi: 10.3390/molecules27227836 (PMC9693348; doi:10.3390/molecules27227836)
Supplement: Supplementary file 1 [file molecules-27-07836-s001.zip › molecules-2022492-supplementary.pdf]

Supporting Information to the paper

Structure Determination of Binuclear Triple-Decker Phthalocyaninato Complexes by NMR via Paramagnetic Shifts Analysis Using Symmetry Peculiarities

Sergey P. Babailov [1], Eugeny N. Zapolotsky [1], Eduard S. Fomin [2], Marina A. Polovkova [3], Gayane A. Kirakosyan [3,4], Alexander G. Martynov [3] and Yulia G. Gorbunova [3,4,*]

[1] Nikolaev Institute of Inorganic Chemistry, The Siberian Branch of the Russian Academy of Sciences, Av. Lavrentyev 3, Novosibirsk 630090, Russia
[2] Institute of Cytology and Genetics of the Siberian Branch of the Russian Academy of Sciences, Av. Lavrentyev 10, Novosibirsk 630090, Russia
[3] Frumkin Institute of Physical Chemistry and Electrochemistry of the Russian Academy of Sciences, Leninskii pr. 31-4, Moscow 119071, Russia
[4] Kurnakov Institute of General and Inorganic Chemistry, Russian Academy of Sciences, Leninskii pr. 31, Moscow 119991 Russia
* Correspondence: yulia@igic.ras.ru

2.2 Paramagnetic NMR shifts analysis

2.2.1 Derivation of calculation expressions

For the case of a polynuclear lanthanide complex, expression (1) can be rewritten as a representation of the total pseudo-contact contribution δ_j^{PC} on the *j*-th nucleus as the sum of independent contributions from each lanthanide Ln(*i*):

$$\delta_j^{PC} = \frac{1}{2N\hbar\gamma}\sum_{i=1}^{Ln}\left[\begin{array}{l}\left(\bar{\chi}^{Ln(i)} - \chi_{zz}^{Ln(i)}\right)\left\langle\frac{1-3\cos^2(\theta_i)}{r_i^3}\right\rangle + \left(\chi_{xx}^{Ln(i)} - \chi_{yy}^{Ln(i)}\right)\left\langle\frac{\sin^2(\theta_i)\cos(2\varphi_i)}{r_i^3}\right\rangle + \\ +2\left(\chi_{xy}^{Ln(i)}\right)\left\langle\frac{\sin^2(\theta_i)\sin(2\varphi_i)}{r_i^3}\right\rangle + 2\left(\chi_{xz}^{Ln(i)}\right)\left\langle\frac{\sin 2(\theta_i)\cos(\varphi_i)}{r_i^3}\right\rangle + 2\left(\chi_{yz}^{Ln(i)}\right)\left\langle\frac{\sin 2(\theta_i)\sin(\varphi_i)}{r_i^3}\right\rangle\end{array}\right] \quad \text{(a)}$$

where r_i, θ_i, φ_i are the spherical coordinates of the *j*-nucleus relative to the *i*-cation Ln(*i*). The use of formula (4) is limited by the complexity of the calculation procedures associated with an increase in the variable parameters (compared to mononuclear lanthanide complexes).

Due to the symmetry of the complexes $Ln_2[(15C5)_4Pc]_3$, where Ln = Tb (**1**) and Dy (**2**) the task for the binuclear complex can be reduced to the quasi-mononuclear case as follows.

Each of the paramagnetic centers Ln(1) and Ln(2), interacting with a set of "paired" protons from the "upper" and "lower" outer decks of phthalocyanine (denoted as α and β), creates pseudo-contact shifts:

$$\delta(\alpha) = \delta^{Ln1}(\alpha) + \delta^{Ln2}(\alpha), \qquad \text{(b)}$$

$$\delta(\beta) = \delta^{Ln1}(\beta) + \delta^{Ln2}(\beta), \qquad \text{(c)}$$

where $\delta^{Ln1}(\alpha)$, $\delta^{Ln2}(\alpha)$ are the pseudocontact contributions to the LIS from the cations Ln(1) and Ln(2) for the proton α, respectively; $\delta^{Ln1}(\beta)$, $\delta^{Ln2}(\beta)$ are the contributions to the LIS from the cations Ln(1) and Ln(2) for the proton β, respectively.

Since Ln(1) = Ln(2), (where Ln(1) = Tb or Dy) and, accordingly, $\delta(\alpha) = \delta(\beta)$, then

$$\delta^{Ln1}(\alpha) + \delta^{Ln2}(\alpha) = \delta^{Ln1}(\beta) + \delta^{Ln2}(\beta) \qquad \text{(d)}$$

From considerations of symmetry it follows that

$$\delta^{Ln1}(\alpha) = \delta^{Ln2}(\beta), \qquad \text{(e)}$$

$$\delta^{Ln2}(\alpha) = \delta^{Ln1}(\beta). \qquad \text{(f)}$$

Then, substituting the obtained equalities into (b), we obtain

$$\delta(\alpha) = \delta(\beta) = \delta^{Ln1}(\alpha) + \delta^{Ln1}(\beta) = \delta^{Ln2}(\beta) + \delta^{Ln2}(\alpha) \qquad \text{(g)}$$

Thus, the structural task is reduced to optimizing the values of $\delta_{calc}(\alpha)$ by analogy with expression (a), which takes the form

$$\delta^{calc}(\alpha) = \sum \chi_{n} * [G^{Ln1}(\alpha) + G^{Ln1}(\beta)] \qquad \text{(h)}$$

Here χ_n (n = 1-5) are the optimized components of the magnetic susceptibility tensor, the values $\frac{(\bar{\chi} - \chi_{zz})}{N \times \hbar \times \gamma}$, $\frac{(\chi_{xx} - \chi_{yy})}{N \times \hbar \times \gamma}$, $\frac{\chi_{xy}}{N \times \hbar \times \gamma}$, $\frac{\chi_{xz}}{N \times \hbar \times \gamma}$, $\frac{\chi_{yz}}{N \times \hbar \times \gamma}$ (expressed in ppm × Å^3) for the quasi-mononuclear complex (five parameters), $G^{Ln1}(\alpha)$ is the coordinate function $G = G(r, \theta, \varphi)$ for the proton α relative to the paramagnetic

center Ln(1), $G^{Ln1}(\beta)$ is the coordinate function $G = G(r, \theta, \varphi)$ for the proton β relative to the paramagnetic center Ln(1).

The agreement parameter *AF* was calculated according to formula (i).

$$AF = \left(\frac{\sum_{i,Ln} W_i (\delta_{LIS} - \delta_{calc})^2}{\sum_{i,Ln} W_i (\delta_{LIS})^2} \right)^{\frac{1}{2}}, \qquad \text{(i)}$$

where W_i is a weighting factor equal to the reciprocal of the square of the experimental error in determining LIS, *AF* is the parameter to be minimized, δ_{LIS} are the experimental values of paramagnetic chemical shifts (CS) (taking into account the diamagnetic contribution), and δ_{calc} are the values of paramagnetic CS calculated as a result of optimization. The adjustable parameters are the components of the magnetic susceptibility tensor in formula (a), and the geometric parameters are set constant.
